# Predictors of long-term HRQOL following cardiac surgery: a 5-year follow-up study

**DOI:** 10.1186/s12955-021-01838-1

**Published:** 2021-08-17

**Authors:** Daiva Grazulyte, Ieva Norkiene, Evaldas Kazlauskas, Inga Truskauskaite-Kuneviciene, Smilte Kolevinskaite, Donata Ringaitiene, Jurate Sipylaite

**Affiliations:** 1 Clinic of Anaesthesiology and Intensive Care, Institute of Clinical Medicine, Faculty of Medicine, Vilnius University, Vilnius, Lithuania; 2 Centre for Psychotraumatology, Institute of Psychology, Vilnius University, Vilnius, Lithuania

**Keywords:** Cardiac surgery, Health-related quality of life, Risk factors, Long-term

## Abstract

**Background:**

The study aimed to evaluate the long-term change of health-related quality of life (HRQOL) and to identify predictors of HRQOL 5 years after cardiac surgery.

**Methods:**

Consecutive adult patients, undergoing elective cardiac surgery were enrolled in the study. HRQOL was measured using the Medical Outcomes Study 36-Item Short-Form Health Survey (SF-36) questionnaire before and 5-years after cardiac surgery. A multivariate latent change modeling approach was used for data analysis.

**Results:**

210 participants (30.5% female) were reached at 5-year follow-up and included in final data analysis. The study revealed, after controlling for gender effects, a significant long-term positive change, in physical functioning (PF, M_*slope*_ = 19.79, *p* < 0.001), social functioning (SF, M_*slope*_ = 17.27, *p* < 0.001), vitality (VT, M_*slope*_ = 6.309, *p* < 0.001) and mental health (MH, M_*slope*_ = 8.40, *p* < .001) in the total sample. Lower education was associated with an increase in PF (M_*slope*_ = 24.09, *p* < 0.001) and VT (M_*slope*_ = 8.39, *p* < 0.001), more complicated surgery (other than the coronary artery bypass graft (CABG) predicted increase in general health (GH, M_*slope*_ = 6.76, *p* = 0.005). Arrhythmia was a significant predictor for lower pre- and post-operative VT and SF.

**Conclusions:**

Overall HRQOL in our sample improved from baseline to five years postoperatively. Further studies including larger patient groups are needed to confirm these findings.

## Introduction

Improving or sustaining Health-related quality of life (HRQOL) is one of the main objectives of modern healthcare. HRQOL incorporates the physical, psychological, and social domains of wellbeing as well as the effects of illness and treatment applied [1]. HRQOL as a measurable outcome is often considered to be one of the most important indicators of advancements in healthcare and disease management resulting in increased life expectancy of those living with chronic health conditions. Moreover, the American Heart Association (AHA) recently defined HRQOL as the “discrepancy between actual and desired functional status and overall impact of health on well-being” and included HRQOL evaluation as a strategic treatment goal for cardiovascular health [2].

Due to its complexity and the aging population, cardiac surgery was always a subject where such outcomes as the rates of postoperative morbidity and mortality were qualified as sufficient in estimating the result of the treatment [3]. Over the last decades, measurement of health outcomes became as important as the patient’s ability to fulfill their psychological, and social needs after the cardiac surgery. Therefore, the monitoring of patients’ HRQOL became one of the strategies to address the holistic concept of health in medical research with the Short-Form Health Survey (SF-36) being among the most popular ways to do it [4]. Heart surgery performed on asymptomatic or mildly symptomatic patients does not usually offer direct or immediate results that can be experienced by an individual. Some patients report lower levels of HRQOL following medical procedures, due to postoperative pain, sleep disturbance, memory problems, and disrupted social interactions by the surgery [5]. On the other hand, among patients with advanced heart failure, surgical treatment might decrease or eliminate symptoms, and significantly improve physical functioning and decrease need for pharmacotherapy, leading to an improvement in overall wellbeing and quality of life [6].

Long-term HRQOL after cardiac surgery or other medical procedures is determined not only by objective changes in health status but by personality and individual behavioral, cognitive, and emotional processes which contribute to adjustment to the changes in health status [7]. Assessment of the long-term HRQOL could provide an insight into how medical procedure or treatment is affecting the psychosocial functioning of the patient over time. Focusing on the patient-centered long-term HRQOL outcome measurements could shift treatment and rehabilitation goals towards multidisciplinary care aimed at improving the overall wellbeing of patients [8]. Recent findings on long-term HRQOL after cardiac surgery are inconsistent and often contradictory. Some studies aimed at investigating the change in HRQOL at one-year follow-up reported improvement in HRQOL while other studies failed to identify any change in HRQOL [5, 9]. Furthermore, few recent studies have explored HRQOL following cardiac surgery at 5 years follow-up or more [3, 10] with promising findings of sustained HRQOL many years after the surgery. Thus, more research addressing long-term HRQOL after cardiac surgery is needed [11].

The present study aimed to evaluate change in HRQOL after 5 years among patients who underwent elective cardiac surgery. Based on previous studies [3, 10], we expected to identify improvement in physical, psychological and social HRQOL outcomes at five years follow-up after the cardiac surgery. Furthermore, we aimed to estimate the role of multiple clinical and psychosocial indicators of long-term HRQOL after cardiac surgery, in particular, dyslipidemia, arrhythmia, hypertension, reduced left ventricular ejection fraction, EuroScore II, type of surgery, age, and education level. We expected these factors to represent the risk of failure to improve the long-term HRQOL after the cardiac surgery.

## Methods

### Patients and data acquisition

In a single-center prospective design study, all consecutive patients undergoing elective cardiac surgery from March of 2013 to April of 2014 at Vilnius University Hospital Santaros clinics were enrolled in a study. Three types of heart surgery were performed: coronary artery bypass grafting (CABG), valve surgery, and complex surgery. Inclusion criteria: patient age ≥ 18 years, Lithuanian language proficiency, the level of cognitive functioning that enabled to respond to the study measures. The first (pre-operative) assessment was performed after admission, prior to surgery. The second assessment was performed via telephone survey 5 years after the surgery. We used five attempts to reach each patient at a 5-year follow-up at various times throughout the day in a two-week period, with one extra call on consecutive 4 weeks. In case of no response, the official records were additionally re-checked and consulted to obtain the patient’s vital status (dead or alive). If the patient was alive and was not reached, he/she was considered as a non-responder and was excluded from the study. Only fully completed questionnaires were included in the analysis. There was no imputation of missing data at the item-level. The study was approved by the Regional Bioethics Committee. Written informed consent was obtained from each patient at inclusion.

### Sample characteristics

**Fig. 1:**
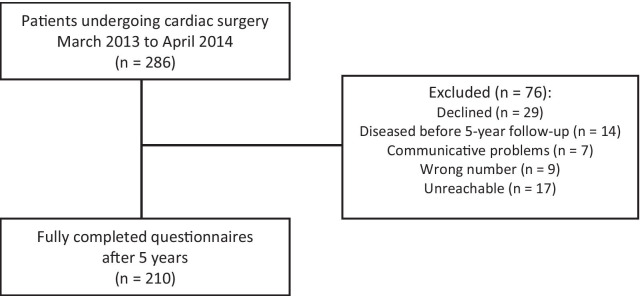
The flow of the study

Out of 286 patients enrolled in the study, 210 adults (30.5% female, aged 19–84 (*M*_age_ = 62.29, *SD*_age_ = 11.29 at the baseline) were reached at 5-year follow-up and were included in data analysis. Excluded from the analyses were 76 patients for the following reasons: declined to participate (n = 29), unreachable (n = 17), diseased prior to 5-year follow-up (n = 14), other reasons (n = 16) (See Fig. 1). Characteristics of the sample are presented in Table 1.

**Table 1:** Sample characteristics at pre-surgery and at 5-year follow-up (N = 210)

	*n*	%
*Sample characteristics at baseline*		
Gender		
Female	64	30.5
Male	146	69.5
Age		
< 70 years	157	74.8
> = 70 years	53	25.2
University degree		
No	146	69.5
Yes	63	30.0
Not disclosed	1	0.5
Dyslipidemia		
No	96	45.7
Yes	114	54.3
Arrhythmia		
No	167	79.5
Yes	43	20.5
Hypertension		
No	43	20.5
Yes	167	79.5
Left-ventricular ejection fraction		
Reduced (< 50%)	74	35.2
Normal (> = 50%)	130	61.9
No information	6	2.9
*EuroScore* II		
Higher mortality risk (> = 2%)	59	28.1
Lower mortality risk (< 2%)	151	71.9
Type of surgery		
Coronary artery bypass graft (CABG)	136	64.8
Other	74	35.2
*Sample characteristics at 5-year follow-up*		
Relationship status		
In a committed relationship	143	68.1
Not in a committed relationship	65	31.0
Not disclosed	2	1.0
Employment		
Employed	58	27.6
Unemployed	75	35.7
Retired	76	36.2
Not disclosed	1	0.5
Residence area		
Urban	154	73.3
Rural	55	26.2
Not disclosed	1	0.5

### Health-related quality of life

The primary outcome measure was Health-related quality of life (HRQOL), measured using the Medical Outcomes Study 36 Item Short Form Health Survey (SF¬36) questionnaire before and 5 years after cardiac surgery [12]. The SF-36 comprises 36 items used to measure 8 domains of health: general health (GH), physical functioning (PF), bodily pain (BP), mental health (MH), role limitations due to physical problems (RP), role limitations due to emotional problems (RE), vitality (VT), and social functioning (SF). Scores of each subscale range from 0 to 100 with higher scores indicating better HRQOL. The SF-36 has been previously used for assessing the HRQOL of cardiac patients in Lithuania [13].

### Clinical and socio-demographic data

For all patients, we collected data on sex, age, relationship status, area of residence (rural vs urban), employment status, level of education, comorbidities (dyslipidemia, arrhythmias, arterial hypertension, left ventricular ejection fraction in %), and type of surgery. New York Heart Association (NYHA) classification, European system for cardiac operative risk evaluation (Euroscore II) [14] was used for assessment of all the patients. Medical and surgical information was collected from an institutional database.

### Statistical analysis

To examine changes in the eight indicators of health-related quality of life (HRQOL) over five years after the cardiac surgery, in comparison to the pre-operative health condition, measured with the same eight indicators, we used a multivariate latent change modeling approach, that provides robust estimates of change over time [15]. We conducted a latent change model on eight parallel processes, in particular, general health (GH), physical functioning (PF), bodily pain (BP), mental health (MH), absence of role limitations due to physical problems (RP), absence of role limitations due to emotional problems (RE), vitality (VT), and social functioning (SF). In the current study, when testing the latent change model with two measurement points, the *intercept* represented the mean level of the HRQOL indicator at Time 1 (before the surgery) and the *slope* represented the change from Time 1 to Time 2 (5 years after the surgery).

As gender has been repeatedly shown to be a significant predictor of HRQOL [16, 17], in the latent change model, we controlled for gender effects on intercepts and slopes of all indicators. To have the latent change model identified, we fixed the residuals and the gender effects above marginal significance (*p* > 0.10) to zero, one by one, until we obtained the final model. Additionally, to identify whether the changes in health indicators were linked with one another and whether the initial levels of GH, PF, BP, MH, RP, RE, VT, and SF were associated with the changes in these indicators, we allowed the correlations between all intercepts and slopes.

After running the multivariate latent change model in the full study sample, we conducted a series of multiple group analyses by testing the preoperative risk factors, in particular, dyslipidemia, arrhythmia, hypertension (yes versus no), reduced left ventricular ejection fraction (LVEF < 50% versus normal EF >  = 50%), and higher EuroScore II (> = 2% mortality risk versus < 2% low-risk group), type of surgery (coronary artery bypass graft only (CABG) versus valve type and complex) as well as psychosocial factors, namely, older age (> = 70 years versus < 70 years old) and higher education (yes versus no) as the moderators of baseline and change rates in HRQOL indicators. To assess possible differences between groups, we assessed differences between the models with fixed versus free parameters of slopes, indicating the same versus different levels of change in health indicators across the groups within moderators. Significant differences between the models with fixed versus free parameters were identified, when at least two of these three criteria were matched: Δχ^2^ significant at *p* < 0.05 [18], ΔCFI (the Comparative Fit Index) ≥ 0.01, and ΔRMSEA (the Root Mean Square Error of Approximation) ≥ 0.015 [19]. To test for differences in HRQOL indicators at Time 1 as well as to compare the change parameters across moderators, we ran the Wald χ^2^ tests.

In all analyses, model fit was evaluated using the Comparative Fit Index (CFI), indicating the discrepancy between the data and the hypothesized model, the Tucker–Lewis Index (TLI), indicating the discrepancy between the chi-squared value of the hypothesized model and the chi-squared value of the null model, and the Root Mean Square Error of Approximation (RMSEA), indicating the discrepancy between the hypothesized model, with optimally chosen parameter estimates, and the population covariance matrix. When evaluating the model fit, we followed the goodness of fit recommendation provided by [20]. In particular, CFI/TLI values higher than 0.90 indicate an acceptable fit, and values higher than 0.95 represent a very good fit; RMSEA values below 0.08 indicated an acceptable fit, and values less than 0.05 suggested a good fit. The analyses were conducted with Mplus 8.2 by using the robust maximum likelihood (MLR) estimator [21].

## Results

The descriptive statistics of the health-related quality of life (HRQOL) indicators at two measurement points and the correlation coefficients across the time points are presented in Table 2. We found that all study variables were approximately normally distributed, as the coefficients of skewness and kurtosis were within the range of ± 2 [22]. Most HRQOL indicators were significantly positively interrelated across the timepoints, except for the pre-operative role limitations due to emotional problems (RE), which was not linked to the RE at 5-year-follow-up.

**Table 2:** Descriptive statistics and correlations of HRQOL indicators across two measurement points

	Pre-surgery (T1)		5-year follow-up (T2)		*t (df)*	*r*
	*M (SD)*	*γ*_*1/*_*γ*_*2*_	*M (SD)*	*γ*_*1/*_*γ*_*2*_		
General health	48.08 (16.36)	0.22/0.34	50.70 (19.79)	0.08/− 0.15	− 1.73 (199)	0.31***
Physical functioning	62.10 (24.31)	− 0.74/− 0.09	76.91 (22.39)	− 1.25/1.16	− 8.23 (204)***	0.39***
Bodily pain	58.35 (24.12)	0.06/− 0.66	60.84 (29.25)	− 0.02/− 0.95	− 1.07 (206)	0.22**
Mental health	64.84 (17.76)	0.03/− 0.97	73.08 (17.17)	− 0.35/− 0.44	− 5.77 (202)***	0.32***
Role limitations (physical)	66.29 (16.80)	0.62/− 0.88	69.34 (28.70)	− 0.56/− 0.73	− 1.28 (197)	− 0.01
Role limitations (emotional)	73.27 (21.06)	0.13/− 1.55	76.23 (25.86)	− 0.70/− 0.66	− 1.42 (206)	0.20**
Vitality	57.58 (19.39)	− 0.01/− 0.48	63.64 (19.48)	− 0.11/− 0.83	− 3.74 (197)***	0.31***
Social functioning	64.43 (26.47)	− 0.33/− 0.77	81.83 (24.86)	− 1.29/0.88	− 7.69 (203)***	0.21**

*M* = mean, *SD* = standard deviation, *γ*_1_ = skewness, *γ*_2_ = kurtosis

^**^p  <  0.01, *** *p * <  0.001

### Changes in health-related quality of life indicators

The multivariate latent change analysis with gender as a control variable yielded a very good model fit (χ^2^ (7) = 3.36, *p* = 0.850, CFI/TLI = 1.000/1.062, RMSEA [90% CI] = 0.000 [0.000, 0.047], SRMR = 0.010). The results indicated significant (*p* < 0.001) positive changes in physical functioning (PF), mental health (MH), vitality (VT), and social functioning (SF) over time. No changes were observed in general health (GH; *p* = 0.067), bodily pain (BP; *p* = 0.248), absence of role limitations due to physical problems (RP; *p* = 0.686), and absence of role limitations due to emotional problems (RE; *p* = 0.169) over the period of 5 years after the heart surgery (see Table 3). For all HRQOL indicators, we found significant negative links (*p* < 0.001) between intercepts and slopes (see Table 3), indicating that lower levels of GH, PF, BP, MH, RP, RE, VT, and SF before surgery were associated with larger increases in these indicators. Significant gender effects were found on intercepts of GH (β_intercept_ = 0.17, *p* = 0.003), PF (β_intercept_ = 0.19, *p* < 0.001), BP (β_intercept_ = 0.19, *p* < 0.001), MH (β_intercept_ = 0.14, *p* = 0.006), VT (β_intercept_ = 0.24, *p* < 0.001), and SF (β_intercept_ = 0.11, *p* = 0.019), indicating higher scores of these HRQOL indicators before heart surgery in men, compared to women. Further, significant gender effects were found on slopes of PF (β_slope_ = − 0.13, *p* = 0.016) and RE (β_slope_ = 0.16, *p* = 0.004), indicating larger increases in physical functioning for men, compared to women and larger decreases in role limitation due to emotional problems in women, compared to men.

**Table 3:** Pearson r correlations of intercepts and slopes of study variables

Intercepts		Slopes							
		1	2	3	4	5	6	7	8
1	General health	− 0.48***	− 0.20***	− 0.07	− 0.12	0.11	− 0.02	− 0.21**	− 0.09
2	Physical functioning	− 0.07	− 0.57***	− 0.07	− 0.08	0.15	− 0.25***	− 0.28***	− 0.28***
3	Bodily pain	0.12	− 0.22**	− 0.55***	− 0.14*	0.14*	− 0.05	− 0.30***	− 0.28***
4	Mental health	− 0.06	− 0.05	− 0.14*	− 0.60***	0.05	− 0.07	− 0.29***	− 0.27**
5	Role limitations (physical)	− 0.06	− 0.09	− 0.08	− 0.11	− 0.51***	− 0.15*	− 0.19**	− 0.12
6	Role limitations (emotional)	− 0.09	− 0.25***	− 0.09	− 0.22**	0.08	− 0.52***	− 0.28***	− 0.18*
7	Vitality	− 0.03	− 0.20**	− 0.18**	− 0.27***	0.13*	− 0.04	− 0.59***	− 0.23**
8	Social functioning	0.12	− 0.15*	− 0.14	− 0.13	− 0.16*	− 0.04	− 0.24**	− 0.67***

*p < 0.05, **p < 0.01, ***p < 0.001

### Moderators of initial levels and changes in health-related quality of life indicators

The intercepts and slopes of the Health-Related Quality of Life (HRQOL) Indicators in the groups representing significant moderators are presented in Table 4. Out of five investigated preoperative factors, only the *arrhythmia* was found to be a significant moderator of the latent change model of HRQOL indicators (Δχ^2^(16) = 29.26, *p* = 0.022, ΔCFI = 0.011, ΔRMSEA = 0.030) with no differences in free versus fixed intercept and slope parameter models testing the moderating effects of dyslipidemia (Δχ^2^(16) = 17.22, *p* = 0.372, ΔCFI = 0.001, ΔRMSEA = 0.012), hypertension (Δχ^2^(16) = 21.83, *p* = 0.149, ΔCFI = 0.002, ΔRMSEA = 0.028), reduced left-ventricular ejection fraction (Δχ^2^(16) = 12.11, *p* = 0.736, ΔCFI = 0.000, ΔRMSEA = 0.000), and higher EuroScore II (Δχ^2^(16) = 19.32, *p* = 0.252, ΔCFI = 0.002, ΔRMSEA = 0.026). The model with all free parameters among arrythmia versus no arrythmia group yielded a very good model fit (χ^2^ (14) = 16.47, *p* = 0.285, CFI/TLI = 0.998/0.962, RMSEA [90% CI] = 0.041 [0.000, 0.107], SRMR = 0.024). The Wald χ^2^ analyses indicated significant differences in initial rates of vitality (VT) (χ^2^ (1) = 10.71, *p* = 0.001) and social functioning (SF) (χ^2^ (1) = 4.03, *p* = 0.045) between the two groups with higher scores of both HRQOL indicators in the no arrhythmia group, compared to the arrhythmia group. No differences between groups were found in terms of change parameters, indicating lower levels of VT and SF in the arrhythmia group at 5 years follow-up after the heart surgery.

**Table 4:** Intercepts and slopes of HRQOL indicators in the full sample and the groups of significant moderators

	Full sample (*N* = 210)		Arrhythmia (no/yes) (*n* = 167/43)		CABG (no/yes) (*n* = 74/136)		Higher education (no/yes) (*n* = 146/64)	
	*M*_*intercept*_	*M*_*slope*_	*M*_*intercept*_	*M*_*slope*_	*M*_*intercept*_	*M*_*slope*_	*M*_*intercept*_	*M*_*slope*_
General health	43.55	2.69	44.45/43.70	2.27/3.36	43.20/44.49	6.76^a^**/0.41^b^	44.20/42.21	1.75/4.39
Physical functioning	53.16	19.79***	54.94/48.38	18.23***/24.45***	56.07/50.52	19.99***/20.07***	49.70^a^/63.96^b^	24.99^a^***/6.49^b^
Bodily pain	51.16	2.69	52.06/50.60	4.07/− 2.80	54.06/50.22	− 0.94/4.71	49.14/59.60	3.81/− 0.18
Mental health	60.68	8.40***	61.85/57.36	8.93***/6.53*	59.20/63.96	7.70**/8.84***	59.12/64.91	9.38***/6.31**
Role limitations (physical)	66.17	1.48	66.61/64.85	− 2.46/3.54	66.26/66.17	5.33/− 6.77	64.86/68.89	2.03/− 8.13
Role limitations (emotional)	73.03	− 4.47	74.33/67.99	− 4.73/− 1.72	76.81/71.09	− 9.25*/0.11	69.77^a^/80.18^b^	− 3.81/− 7.11
Vitality	50.36	6.30***	53.86^a^/40.66^b^	6.10**/6.80*	50.91/48.93	8.28**/5.21**	49.41/52.38	8.39^a^***/1.71^b^
Social functioning	59.68	17.27***	62.95^a^/50.39^b^	15.68***/23.30***	61.64/59.16	13.81***/19.10***	58.76/63.81	17.35***/17.49***

*M* = mean, *p < 0.05, ** *p* < 0.01, ***p < 0.001

^a,b^Different letters indicate statistically significant differences among groups

The *type of surgery* (coronary artery bypass graft (CABG) versus complex/valve surgery) was also found to be a significant moderator of the latent change model of HRQOL indicators (Δχ^2^(16) = 30.866, *p* = 0.014, ΔCFI = 0.011, ΔRMSEA = 0.065). The model with all free parameters among the two groups yielded a very good model fit (χ^2^ (14) = 12.36, *p* = 0.577, CFI/TLI = 1.000/1.027, RMSEA [90% CI] = 0.000 [0.000, 0.084], SRMR = 0.022). The Wald χ^2^ test indicated significant differences in change rates of general health (GH) (χ^2^ (1) = 4.41, *p* = 0.036) among the CABG versus other types of surgery groups. The complex/valve group reported a significant increase in GH, while change in GH was not observed in the CABG group. However, complex/valve group reported a significant decrease in absence of role limitations due to emotional problems (RE) with no changes of RE in CABG group over the period of 5 years after the heart surgery. It should be noted that the difference in the change rates of RE among the two groups was not supported by the Wald χ^2^ test (χ^2^ (1) = 2.22, *p* = 0.137).

Older age (> = 70 years versus < 70 years old) was found to have no moderating effects on the latent change model of HRQOL indicators (Δχ^2^(16) = 13.96, *p* = 0.602, ΔCFI = 0.000, ΔRMSEA = 0.000) when the *higher education* yielded a significant moderating effects (Δχ^2^(16) = 29.64, *p* = 0.020, ΔCFI = 0.009, ΔRMSEA = 0.060). The model with all free parameters among higher education versus other type of education groups yielded a very good model fit (χ^2^ (14) = 11.56, *p* = 0.642, CFI/TLI = 1.000/1.040, RMSEA [90% CI] = 0.000 [0.000, 0.079], SRMR = 0.021). The Wald χ^2^ analyses indicated significant differences in change rates of physical functioning (PF) (χ^2^ (1) = 11.52, *p* < 0.001) and vitality (VT) (χ^2^ (1) = 4.05, *p* = 0.044) between two groups with the significant increase of both HRQOL indicators in the lower education group and stability in the higher education group. However, the higher education group reported significantly higher scores of PF before the surgery, compared to the lower education group (χ^2^ (1) = 3.97, *p* = 0.046), while initial VT scores were found to be equal among groups. However, the lower education group reported significantly lower scores in role limitations due to emotional problems (RE) before the surgery, compared to the higher education group (χ^2^ (1) = 10.56, *p* = 0.001) with no differences in change rates between groups, indicating higher levels of RE in the higher education group at 5-year follow-up.

## Discussion

The main aim of the current study was to assess changes in the physical and mental health of patients following cardiac surgery over a period of five years after the surgery. The results from this study support previous research [1, 5, 8, 23] that cardiac surgery may improve functional mobility, can have a long-lasting positive effect on social independence and overall higher quality of life. Based on the findings of the current study, patients experienced HRQOL as significantly improved 5 years after cardiac surgery, this reflected by increases in self-assessed HRQOL domains, in particular, physical health, mental health, vitality, and social functioning on the SF-36. It is worth mentioning, that our results support a the link between improvement in physical condition after heart surgery and higher self-perceived mental and social functioning over five years. These results suggest that the integration of mental and physical health assessment could result in a more holistic approach in the understanding of postoperative recovery.

Longitudinal studies assessing the long-term trajectories of postoperative HRQOL highlight the effects of natural aging [10]. In our study, however, HRQOL changes were observed in both elderly and younger patients. The more significant improvement in HRQOL 5 years after surgery was achieved in patients with lower preoperative quality of life, meaning that those with relatively better preoperative HRQOL may underestimate the positive effects of the surgery. This paradoxical effect in deterioration of health perception despite favorable surgical results was observed by previous authors [24, 25]. Immediate postoperative deterioration in HRQOL might be a result of increased pain, reduced mobility, and increased social dependence. However, the long-term interactions between self-perceived preoperative and postoperative health could be related to overall illness beliefs, which play an important role in recovery, engagement in follow-up, and postoperative rehabilitation [26, 27]. We suggest that identification and addressing of patient maladaptive illness beliefs before surgery could positively affect outcomes and perception of recovery.

Previous studies exploring associations between patient education level and post-procedural outcomes concluded that education level and HRQOL have an inverse relationship [23]. Similarly, in our study, postoperatively overall improvement in the perception of health was more prominent in patients with lower education levels. Our data suggest that patients with higher education experienced less restriction in social activities because of emotional problems preoperatively (RE). We suggest that patients with higher education had better coping skills in everyday activities and therefore were functioning better before cardiac surgery and experienced fewer changes in HRQOL at a 5-year-follow-up. This suggestion is supported by significantly higher HRQOL values in the higher education subgroup pre-operatively. The role of education should be evaluated in the context of other socioeconomic variables such as income, living conditions, or employment. Further research is needed to determine the impact of educational level on outcomes.

Our findings are consistent with previous reports indicating substantial gender-related disparity in physical HRQOL both before and after cardiac surgery with women having lower scores in several HRQOL subdomains [16]. We controlled for gender in analyzing change and predictors of HRQOL in our study, however, due to a rather small proportion of female participants in our study, we did not examine the effects of gender on HRQOL. Future studies should explore the moderator effects of gender on long-term HRQOL.

Our study suggests that patients with increased preoperative risk, as evidenced by the high Euroscore II values, underwent postoperative recovery as successfully as the lower risk group, and showed significant improvement in their self-perceived physical health, mental and social functioning. Patients after low-risk CABG surgery experienced no improvement in general health (GH), while improvement in GH was observed in patients after higher-risk surgeries. Further research is needed to determine the factors that may assist successful recovery in high-risk patients and influence the perception of wellbeing postoperatively. We have also identified some differences in HRQOL in patients with the presence of atrial fibrillation (AF). In particular, we found lower levels of vitality and social functioning in patients with arrhythmia at baseline and five years after the surgery. Studies using HRQOL outcomes confirm that patients diagnosed with AF experience more functional limitations, depression, anxiety, and distress [27]. Lower HRQOL and more mental health problems might contribute to reduced adherence to medications and engagement in physiotherapy, and eventually have an impact on physical recovery after surgery [28]. The mutual relation between anxiety and AF initiation and progression remains ambiguous. Cochrane systematic review suggests comprehensive behavioral interventions for patients with atrial fibrillation [27]. Our data demonstrated that more complex strategies should be implemented to enhance the functional recovery of patients with AF, complemented by the implementation of educational and behavioral interventions, in particular self-care and self-management programs.

### Limitations

This is one of the first studies to explore long-term HRQOL in a 5-year prospective study. While study findings are promising several limitations needs to be addressed. First, the current study sample was restricted to the collection of data in a single site and a comparably small sample size that limited the possibility to identify the subgroups of patients with different patterns of clinical outcomes. Replication of study in larger samples and applying the person-oriented approach in the analyses is needed in the future. Second, around 27% dropout rate could have impacted our findings, with patients with more severe medical conditions and lower HRQOL potentially among participants who did not respond and participate. Third, we rely on self-reported HRQOL in our study. While the SF-36 is a widely used measure for HRQOL additional medical examination, some recent studies indicated that other measures could be more appropriate for assessing specific patient-reported outcomes and may be more responsive to change in HRQOL [29, 30]. Therefore, at follow-up the inclusion of other specific patient-reported outcomes could have provided more insight into the study.

## Conclusions

Our findings demonstrated a positive change in HRQOL 5 years after cardiac surgery. Overall in our study sample, HRQOL improved from baseline to five years after cardiac surgery. Moreover, an improvement was more significant in patients demonstrating lower rates of SF-36 before the surgery. The study provides insights for patient care as it shows that multiple factors, including psychosocial functioning, contribute to recovery and quality of life after medical procedures, and improvement cannot be predicted solely by the clinical status of the patient or the medical procedures.

## Data Availability

Data supporting the findings of the study could be obtained from the corresponding author upon request.
